# The Impact of Marangoni and Buoyancy Convections on Flow and Segregation Patterns during the Solidification of Fe-0.82wt%C Steel

**DOI:** 10.3390/ma17051205

**Published:** 2024-03-05

**Authors:** Ibrahim Sari, Menghuai Wu, Mahmoud Ahmadein, Sabbah Ataya, Nashmi Alrasheedi, Abdellah Kharicha

**Affiliations:** 1 Metallurgy Department, Montanuniversitaet of Leoben, Franz-Josef-Str. 18, A-8700 Leoben, Austriaabdellah.kharicha@unileoben.ac.at (A.K.); 2 Department of Production Engineering and Mechanical Design, Tanta University, Tanta 31512, Egypt; 3 Mechanical Engineering Department, Imam Mohammad Ibn Saud Islamic University, Riyadh 11564, Saudi Arabia

**Keywords:** solidification, steel alloy, segregation, surface tension, Marangoni flow, Rayleigh–Bénard–Marangoni instability

## Abstract

Due to the high computational costs of the Eulerian multiphase model, which solves the conservation equations for each considered phase, a two-phase mixture model is proposed to reduce these costs in the current study. Only one single equation for each the momentum and enthalpy equations has to be solved for the mixture phase. The Navier–Stokes and energy equations were solved using the 3D finite volume method. The model was used to simulate the liquid–solid phase transformation of a Fe-0.82wt%C steel alloy under the effect of both thermocapillary and buoyancy convections. The alloy was cooled in a rectangular ingot (100 × 100 × 10 mm^3^) from the bottom cold surface to the top hot free surface by applying a heat transfer coefficient of h = 600 W/m^2^/K, which allows for heat exchange with the outer medium. The purpose of this work is to study the effect of the surface tension on the flow and segregation patterns. The results before solidification show that Marangoni flow was formed at the free surface of the molten alloy, extending into the liquid depth and creating polygonized hexagonal patterns. The size and the number of these hexagons were found to be dependent on the Marangoni number, where the number of convective cells increases with the increase in the Marangoni number. During solidification, the solid front grew in a concave morphology, as the centers of the cells were hotter; a macro-segregation pattern with hexagonal cells was formed, which was analogous to the hexagonal flow cells generated by the Marangoni effect. After full solidification, the segregation was found to be in perfect hexagonal shapes with a strong compositional variation at the free surface. This study illuminates the crucial role of surface-tension-driven Marangoni flow in producing hexagonal patterns before and during the solidification process and provides valuable insights into the complex interplay between the Marangoni flow, buoyancy convection, and solidification phenomena.

## 1. Introduction

In many industrial sectors, particularly those involving casting processes, temperature gradients and free surface phenomena are prevalent. These conditions are often induced by specific cooling walls, resulting in nonuniform temperature distributions within the casting molds. The temperature gradient induces flow instability due to density variations and gravity. These instabilities are collectively referred to as Rayleigh–Bénard (RB) instabilities. In the presence of a free surface, another instability arises, driven by surface tension variations. This phenomenon is known as Bénard–Marangoni (BM) instability. However, Rayleigh–Bénard–Marangoni (RBM) instability encompasses situations where both effects are coupled. The well-known Rayleigh–Marangoni numbers are dimensionless numbers that have an important role in analyzing the behavior of fluids that are driven by buoyancy and the surface tension gradient. These two dimensionless numbers are important in RBM convection in predicting a fluid flow regime and in characterizing the relative effects of surface tension and viscous forces. Numerous authors have developed several numerical and experimental studies, taking into account RBM instability [1,2,3,4,5,6]. Rachid Es Sakhy et al. [7] conducted a numerical investigation of RBM flow in a cylindrical geometry containing silicon oil with a free upper surface which was heated from the bottom. This configuration generated a temperature gradient that led to the formation of convective cells. Moreover, they analyzed the formation of cells and hexagonal patterns during flow for different Marangoni and Rayleigh numbers. The authors claimed that the formation of hexagonal cells was directly related to the Marangoni number. However, in the absence of surface tension (Ma = 0), no hexagonal patterns were formed. Thus, the Marangoni number was fixed at a rather high value, Ma = 2000, and hexagon cells were formed, and the number of the cells increased with the increase in the Rayleigh number. In addition, the authors reported that the size and the number of the hexagonal cells increased with the increase in the Marangoni number. S. Rahal et al. [8] experimentally studied the dynamics pattern and the free surface deformation during Bénard–Marangoni convection in a small circular geometry of six aspect ratio, which contained a horizontal layer of silicone oil. They considered the effect of various physical parameters, such as Ma, Pr, and Biot numbers, on the formation and dynamics of Bénard–Marangoni patterns, which were in the form of polygonal cells. The authors reported that the number of cells decreased as a function of the Ma number. Also, the cells were found to be larger for a higher Pr number than for lower values. However, the number of cells increased as a function of the Biot number. Marc Medale and Pierre Cerisier [9] conducted a numerical investigation on the coupled effect of the gravity- and capillarity-driven thermo-convection of a fluid layer of silicon oil heated from below (Bénard–Marangoni convection). The authors explored various container shapes, including triangular, quadrilateral, pentagonal, hexagonal, and circular. Their findings revealed a noteworthy correlation: an increase in the aspect ratio led to a proportional increase in the number of hexagonal cells observed in the convective flow pattern. To substantiate the accuracy of their numerical simulations, the numerical results were compared against the experimental data obtained by Koschmieder and Prahl [10]. The validation process showcased a good agreement between the numerical predictions and the experimental measurements, enhancing the reliability of the outcomes study. Bobach et al. [11] used the particle finite element method (PFEM) to simulate the phase-change process, taking into account the surface tension. The authors presented a series of test cases to validate their simulation method with temperature-driven convective flows in 2D. The authors reported that the PFEM was suitable for free surface deformation, despite the free surface flow not being included in their work. In addition, their model successfully captured the effect of Marangoni and natural convections, which are the driving forces of the flow. Their simulation results were in good agreement with the experimental data. Mendis et al. [12] proposed a 3D numerical simulation model to investigate the effect of solutal Marangoni convection on flow instabilities in the presence of thermal Marangoni convection in a Si-Ge liquid bridge. Different aspect ratios were investigated for a disk with cold top and hot bottom surfaces under zero gravity. The authors claimed that the critical Marangoni number decreased with the increase in the aspect ratio; however, the obtained simulation results displayed the same tendency as the previous results did in the case of pure thermal Marangoni convection. Rezaee et al. [13] introduced and investigated a 3D model of flow in microscale foams within a geometry containing plateau borders (PB), nodes, and films to simulate the recirculation of the Marangoni flow for different interfacial mobilities. As a result, thicker films are significantly more effective in reducing Marangoni flow compared to PB flow. Additionally, researchers further investigated the impact of the thickness and surface tension mobility on the velocity of Marangoni flow. Furthermore, they validated their model using previous experimental and analytical studies. Hooshanginejad et al. [14] conducted experiments to explore the dynamics (instability) of water droplets on glycerol–water solutions. They observed the formation of fingering patterns that were developed from the interplay between outward buoyancy and inward Marangoni flow. These findings offer insights into phenomena known as the buoyancy–Marangoni fingering of a miscible spreading drop phenomena.

In the present paper, a 3D numerical simulation model is proposed to investigate the role of Marangoni flow on the formation of hexagonal flow and segregation structures during the solidification of Fe-0.82wt%C steel alloy. Particular attention has been directed towards regulating both the size and the number of the formed hexagonal cells. The movement of the fluid was driven by the density variations of the fluid caused by the temperature and concentration gradients, known as the buoyancy effect, and by the surface tension gradient, which is known as Marangoni-driven flow. The outline of this paper is as follows: Firstly, a brief description is provided of the mathematical model and the physical configuration (including governing equations, boundary conditions, and assumptions) used in the study. Then, a comprehensive discussion of the obtained results pertaining to flow patterns, temperature distribution, solid fraction, and segregation structures is presented. The influence of liquid layer thickness will also be thoroughly examined. Finally, a general conclusion highlights the key findings of this study.

## 2. Numerical Model

A multiphase mixture model is proposed to simulate the liquid–solid phase transformation of Fe-0.82wt%.C steel alloy in the presence of Rayleigh–Bénard–Marangoni (RBM) convection. This model draws inspiration from the previous work of Wu and Ludwig [15,16], with a key distinction: the concept of the mixture is applied only to momentum and enthalpy. The present model incorporates essential features used by Yong Tang et al. [17], encompassing solute redistribution (as described by the liquid transport equation), among others. However, the two considered phases are distinctly identified as the liquid (primary phase) and the solid (secondary phase). Their volume fractions sum up to one, denoted as $f_{l}{+f}_{s}=1$. The solid phase in this model represents globular columnar structures. The mixture phase is an artificial construct representing a combination of liquid and solid phases. Its properties are determined by the relative proportions of the liquid and solid phases, known as the phase fraction. In this model, thermodynamic equilibrium is assumed to exist at the interface between the solid and liquid phases. Additionally, a single temperature is assigned to the mixture within each control volume. The general form of the continuity equation for the mixture is defined in Equation (1), as defined by Yong Tang et al. [17].

(1) $$\frac{\partial \rho }{\partial t}+\nabla \cdot \left(\rho {\vec{u}}_{m}\right)=0$$

where $\rho =\rho_{m}=f_{l}\rho_{l}+f_{s}\rho_{s}$ is the mixture density and ${\vec{u}}_{m}=\frac{f_{l}\rho_{l}{\vec{u}}_{l}+f_{s}\rho_{s}{\vec{u}}_{s}}{\rho_{m}}$ is the mixture velocity. However, in the present work, the density in the liquid and the solid phases is considered constant ($\rho_{l}=\rho_{s}$); it will be referred to as “$\rho_{0}$”, except in the gravitational force, which will be discussed later. Also, the solid velocity was considered to be zero (${\vec{u}}_{s}=0$), as the solid structure is considered to be columnar. The drift velocity is determined by the slip velocity between the two phases, which is also known as the relative velocity, ${\vec{u}}_{l-s}={\vec{u}}_{s}-{\vec{u}}_{l}$ (with ${\vec{u}}_{s}=0$).

The Navier–Stokes equation describes the momentum conservation, as defined in Equation (2):

(2) $$\frac{\partial }{\partial t}\rho_{0}{\vec{u}}_{m}+\nabla \cdot \left(\rho_{0}{\vec{u}}_{m}{\vec{u}}_{m}\right)=-\nabla p+\nabla \left(\rho_{0}\mu \nabla {\vec{u}}_{m}\right)+\rho \vec{g}-{\vec{F}}_{Darcy}$$

The last term on the right-hand side of Equation (2) is the momentum sink due to the reduced porosity in the mushy zone define by the Carman–Kozeny function and it takes the following form:

(3) $${\vec{F}}_{Darcy}=\frac{{f_{s}}^{2}}{{\left(1-f_{s}\right)}^{3}+\epsilon }A_{mush}{\vec{u}}_{l}$$

where $A_{mush}=10^{7}$ kg m^−3^ s^−1^ is the mushy zone coefficient, $\epsilon =10^{-3}$ is an arbitrary constant which was used to avoid dividing by zero (Samara et al. [18]; Robynne and Groulx, [19]), and $f_{s}\;$is the solid frcation defined in Equation (4).

(4) $$f_{s}=\frac{f_{s}^{n-1}C_{s}^{*n-1}+\left(1-f_{s}^{n-1}\right)C_{l}-C_{l}^{*}}{\left(C_{s}^{*}-C_{l}^{*}\right)}$$

where $c_{l}^{*},c_{s}^{*}$ are the equilibrium liquid and solid solute concentration at the liquid–solid interface, respectively, and (n − 1) refers to the previous time step. The liquid concentration, $C_{l}$, is given in Equation (9). The solute concentrations at interface, $c_{l}^{*}$ and $c_{s}^{*}$, are expressed by Equation (5), where $T_{M}\;$is the melting temperature of pure iron, k is the partition coefficient, and m_l_ is the liquidus slope.

(5) $$c_{l}^{*}=\left(T-T_{M}\right)/m_{l},c_{s}^{*}=kc_{l}^{*}$$

Except for the density and the surface tension that are defined in Equations (6) and (7), respectively, all other thermophysical properties are treated as constants. However, the Boussinesq-type approximation was only applied to the gravitational force, resulting in density variations with temperature and concentration (as specified in Equation (6)). Additionally, surface tension was identified as a temperature-dependent function, as defined in Equation (7).

(6) $$\rho =\rho_{0}\left(1+\beta_{T}∆T+\beta_{c}∆C\right)$$

(7) $$\sigma \left(T\right)=\frac{\partial \sigma }{\partial T}\left(T-T_{liq}\right)$$

where $\beta_{T}$ and $\beta_{c}$ are the thermal and solutal expansion coefficients. The temperature coefficient of the surface tension is denoted by $\frac{\partial \sigma }{\partial T}>0$, and it was applied to the free top surface, assuming that surface tension increases with temperature.

The enthalpy conservation equation for the mixture is defined in Equation (8), as follows [17]:

(8) $$\frac{\partial }{\partial t}\left(\rho_{0}f_{l}c_{pl}T_{l}+\rho_{0}f_{s}c_{ps}T_{s}\right)+\nabla \cdot \left(\rho_{0}f_{l}{\vec{u}}_{l}h_{l}+\rho_{0}f_{s}{\vec{u}}_{s}h_{s}\right)=\nabla \cdot \left(k_{eff}\nabla T\right)+\rho_{0}L\frac{\partial f_{s}}{\partial t}$$

where $k_{eff}=f_{l}k_{l}+f_{s}k_{s}$ is the effective thermal conductivity of the mixture phase; $k_{l}\;$and $k_{s}\;$are the thermal conductivities of the liquid and solid, respectively; “L” is the latent heat of fusion. $T_{l}$, $h_{l}$, $T_{s}$, and $h_{s}$ are the liquid temperature, enthalpy, and solid temperature and enthalpy, respectively.

The species conservation equation for the liquid phase (liquid transport equation) is given in Equation (9) [20]:

(9) $$\frac{\partial }{\partial t}\left(\rho f_{l}c_{l}\right)+\nabla \cdot \left(\rho f_{l}{\vec{u}}_{l}c_{l}\right)=\nabla \cdot \left(\rho f_{l}D_{l}\nabla c_{l}\right)+C_{sl}$$

where $C_{sl}=-\rho_{0}\frac{\partial c_{s}^{*}f_{s}}{\partial t}$ denotes the species exchange rate between the solid and liquid phases. 

The mixture concentration of the liquid and the solid phases is presented in Equation (10), as follows:

(10) $$c_{mix}=f_{l}c_{l}+f_{s}c_{s}^{*}$$

## 3. Simulation Setup

**Figure 1:**
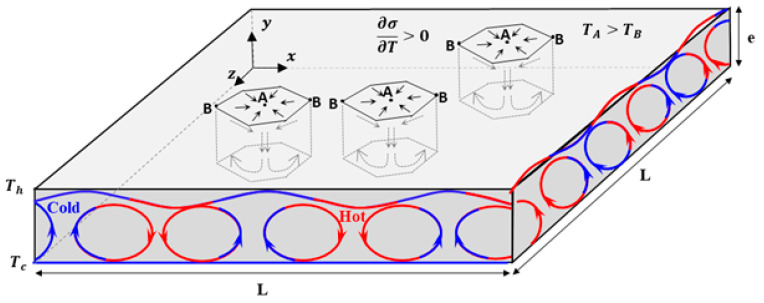
Sketch of the simulated domain of the solidification ingot.

The simulation domain is represented in Figure 1 as a shallow rectangular ingot of thickness “e” and length “L” filled with molten Fe-0.82wt%C alloy. The side surfaces are assumed to be adiabatic; meanwhile, the bottom and the top surfaces have cold and hot temperatures, noted as T_c_ and T_h_, respectively. The top surface is treated as a free surface in contact with air. Therefore, a positive coefficient of the surface tension is applied on the top surface, which means that the surface tension increases with increases in the temperature. The nonuniform temperature at the top surface (induced by the surface tension) and within the ingot thickness (induced by the vertical temperature gradient) leads to the formation of original flow patterns and convective cells, as depicted in Figure 1. The direction of the flow within these cells is related to the temperature coefficient of the surface tension $\frac{\partial \sigma }{\partial T}$, which will be discussed later. 

The deformation of the free surface can generally be neglected if the Crispation number (Cr=μα/σe≪1) is too small and the Galileo number ($Ga=ge^{3}/\nu \alpha \gg 1$) is too large [4], where μ,α, σ,e, and g denote the fluid viscosity, thermal diffusivity, surface tension, liquid thickness, and the acceleration due to gravity, respectively. This hypothesis is applicable to the current case study because $Cr=7.6\times 10^{-4}$ and $Ga=2.1\times 10^{6}$. Therefore, the free surface was considered flat. The thermophysical properties of Fe-0.82wt%C are given in Table 1.

## 4. Results and Discussion

The mixture multiphase model introduced in Section 2 was implemented in the 3D rectangular geometry described in Section 3. In Section 4.1, the effects of thermal gradient and liquid depth on cell formation, examining the number and size of cells, will be presented. Section 4.2 explores the influence of the positive surface tension coefficient on flow direction. The impact of Marangoni on flow and temperature is presented in Section 4.3, while Section 4.4 investigates the role of Marangoni during solidification, focusing on the solid fraction and segregation patterns.

### 4.1. Effect of Liquid Depth and Thermal Gradient on Flow Pattern

The flow motion arises from the interplay between thermocapillary forces (induced by temperature-dependent surface tension) and buoyancy forces (driven by density variations due to temperature and concentration gradients). While the gravity vector acts from top to bottom, it is crucial to note that the primary driver of hydrodynamic instability is the Marangoni flow, which is formed at the free surface of the molten alloy, extending into the liquid depth. The Marangoni flow interacts with the natural convection, forming flow instability known as Rayleigh–Benard–Marangoni (RBM) instability. However, the solutal buoyancy is absent in Section 4.1, Section 4.2 and Section 4.3, as the liquid prior to solidification preserves its initial concentration of 0.82wt%C. Three-dimensional simulations are developed to investigate the impact of the liquid depth (thickness) and the thermal gradient on the formation of Marangoni cells. Calculations were conducted on three cases with configurations given in Table 2. All the studied flow situations are steady. However, the results have been evaluated using a transient solver. 

The important flow parameters for RBM convection are the Marangoni and Rayleigh numbers, which are defined by Equations (11) and (12), as follows:

(11) $$Ma=\frac{\partial \sigma }{\partial T}\frac{e\;DT}{\mu \alpha }$$

(12) $$Ra=\frac{g\beta_{T}{\frac{e}{L}}^{4}DT}{\nu \alpha }$$

These two dimensionless numbers are important in RBM convection. Rachid Es Sakhy et al. [7] analyzed the effect of the Marangoni number on flow for different Marangoni and Rayleigh numbers. They found that there are no hexagonally shaped convection cells formed for different Rayleigh numbers or Ma = 0. Nevertheless, as they set the Marangoni number to a high value (Ma = 2000), hexagonal cells were initiated and their number increased with an increasing Rayleigh number. In addition, the authors reported that the size and the number of hexagonal cells increase with an increase in the Marangoni number. However, the size and the number of the cells might also be changed, depending on the alloy type.

**Figure 2:**
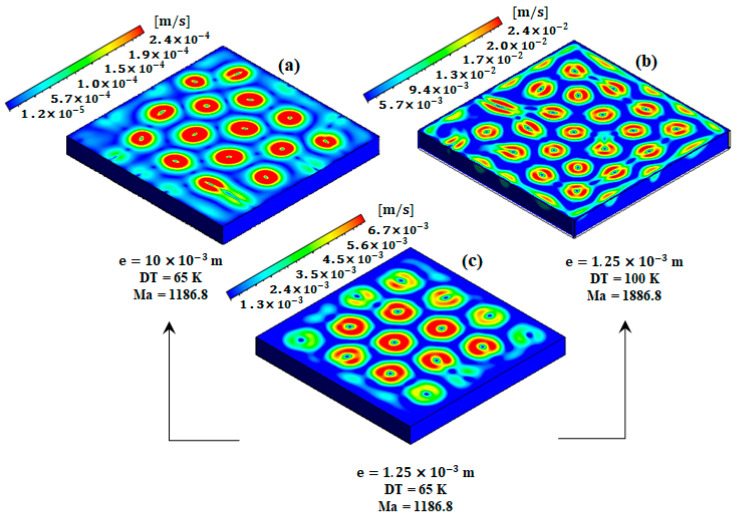
Velocity field for different configurations of geometry and thermal gradient. The thermal gradient and the ingot thickness for cases (**a**–**c**) are DT = 65, 100, 65 K and e = 10, 1.25, and 1.25 mm, respectively.

Figure 2 shows the velocity fields driven by RBM flow before solidification, where the Fe-0.82wt%C alloy is completely in a liquid state. The flow patterns were formed in hexagonal shapes due to the effect of the surface tension, which will be discussed in the next section. As has been found in numerous studies [3,7,8,9,10], the formation of the hexagonal cells is mainly caused by the Marangoni flow, driven by the surface tension. However, in [6,7,10], the authors reported that the size and the number of the hexagons is directly affected by the flow configuration and the aspect ratio. In order to examine the effect of the thermal gradient on flow configuration, Figure 2b,c present two cases that have the same aspect ratio but different thermal gradient (DT = 65 K and 100 K, respectively). In these cases, the number of convective cells increased from 15 to 21 cells with the increase in the thermal gradient from 65 K to 100 K, which consequently increases the Marangoni number from Ma = 1186.8 to 1825.9. Thus, six more cells were added as a result of increasing the thermocapillary forces. However, the size of the formed cells decreased. In addition, the velocity value increased by about 3.5 times with the increase in the thermal gradient. The temperature coefficient of the surface tension was set to $\frac{\partial \sigma }{\partial T}=5\times 10^{-4}$ N/m/K for the cases of low thickness (case b and c). To assess whether Marangoni values are within the magnitude of real steel melt, we would typically need experimental data. These data would include measurements or calculations related to the surface tension gradients and the associated Marangoni effect for molten steel. Due to the unavailability of these data, a parametric study was devoted to defining the corresponding value of this coefficient for a steel alloy. Based on the prescribed model, the value of $\frac{\partial \sigma }{\partial T}=5\times 10^{-4}$ N/m/K can predict the effect of Marangoni flow on the formation of hexagonal patterns, which suggests this is the actual value of the Fe-0.82wt%C alloy. However, no validation has been made to confirm this judgement.

To examine the effect of the ingot thickness on the formation of the hexagonal cells, a larger geometry (8-fold) is presented in case (a) in Figure 2. To keep the same Marangoni number (Ma = 1186.8), the value of $\frac{\partial \sigma }{\partial T}$ was reduced to $6.25\times 10^{-5}$ N/m/K (i.e., eight times less), while the thermal gradient remained unchanged (DT = 65 K). Based on the results of cases (a) and (c), with increasing the thickness to 8-fold, the number of the cells remained the same (15 cells), but their sizes increased dramatically with the increase in the thickness. According to these analyses, the general trend is that the number of Marangoni cells increases with an increasing Marangoni number. However, with increasing the thickness and keeping the same Marangoni, the number of cells remained the same, but the cell size increased. The flow strength was influenced by the characteristic length, which impacted the flow dynamics. Variations in the computing domain size led to changes in the corresponding Rayleigh and Marangoni numbers and then alterations in the flow characteristics. However, the case of e = 1.25 mm was investigated in this part (Figure 2b,c) to understand the effect of the thermal gradient and the liquid depth on the formation of hexagonal patterns. However, for the next investigations, only the geometry with e = 10 mm (see Figure 1) will be considered.

### 4.2. Effect of Surface Tension on the Flow Direction of Liquid

In this section, the flow pattern at the free surface before the solidification is investigated. Numerous mesh refinements were tested to examine the convergence of the model implemented and the minimal mesh size, which ensures the mesh’s independence of the given solution. The transport equations discussed in Section 2 were solved using the pressure–velocity coupling SIMPLEC method within the finite volume method. To achieve convergence, up to eight iterations were performed for each 0.01 s timestep, to reduce the normalized residuals. The convergence criteria for the continuity equations, the momentum equations, and the solute equations were all set to 10^−3^, while 10^−6^ was chosen for the enthalpy equations (Equation (8)).

**Figure 3:**
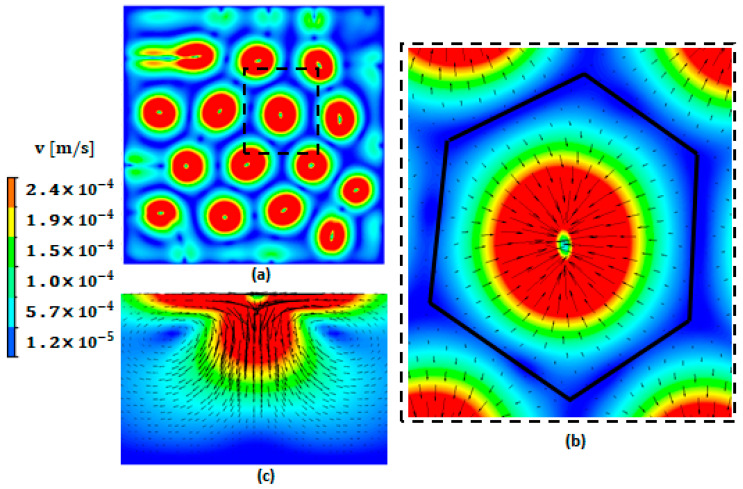
Hexagonal patterns produced by Marangoni effect at the free top surface: (**a**) taken before the solidification; (**b**) zoomed-in view of one hexagon inside the dashed square; (**c**) velocity vector and colored magnitude level inside one hexagon at the vertical cut plane. Ma = 1186.8 and Ra = 2615.5.

Figure 3 represents the velocity field (colored magnitude levels and black vectors) at the free surfaces (a) and (b) and on the cross-section presented in (c). The Marangoni effect is the mass transfer along an interface between two fluids due to the surface tension gradient (fluid from areas with low surface tension is transferred to areas with higher surface tension) [23]. The Marangoni flow dominates the flow within the hexagonal cells (Figure 3a). However, a downward flow is generated by RBM convection at the centers of the hexagonal cells, as shown in Figure 3c. According to [23], the sign of the temperature gradient of the surface tension ($\frac{\partial \sigma }{\partial T}$) determines the direction of the fluid flow at the surface tension. A negative value of $\frac{\partial \sigma }{\partial T}$ indicates that the surface tension (σ) reduces with increasing temperature, prompting a radial outward flow. Conversely, a positive surface tension gradient leads to a radial inward flow, as shown schematically in Figure 3, which is the case in the present study.

### 4.3. Effect of Marangoni on Flow and Temperature Fields before Solidification

**Figure 4:**
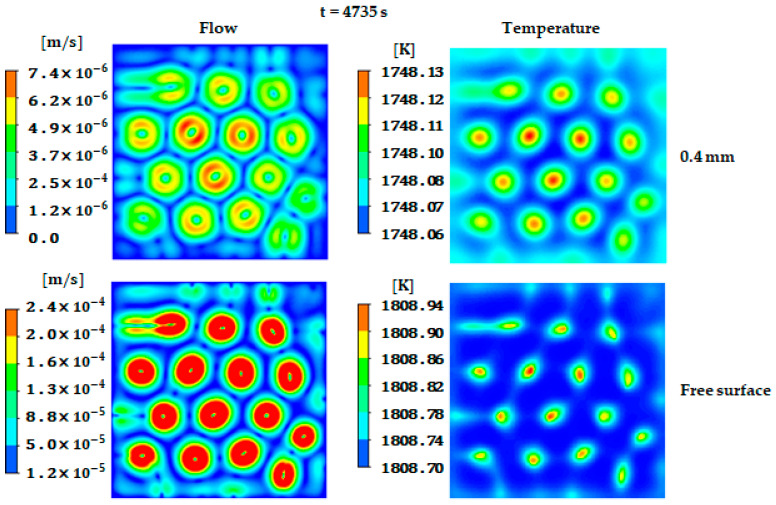
Velocity (**left** column) and temperature (**right** column) fields at the free surface and e = 0.4 mm horizontally cut plane (*xz* plane).

In this section, the effect of Marangoni convection on the flow and temperature distribution before solidification is presented. The resulting fields of the flow and temperature are shown in Figure 4 in the left and right columns, respectively. The results at the horizontally cut plane of e = 0.4 mm from the bottom (Figure 4 top) are also compared to those at the free surface (Figure 4 bottom). As discussed earlier, the nonuniform temperature distribution exists along the free surface, which introduces a gradient in the surface tension. Despite the weak temperature difference between the hot and the cold regions along the free surface (~0.24 K), it was sufficient to direct the liquid metal from the cold towards the hot regions as a result of the positive temperature coefficient of the surface tension.

The movement of the flow along the free surface leads to the birth of fifteen convective hexagonal cells. Marangoni flow extended vertically along the liquid depth toward the bottom, producing regular, steady, hexagonal flow patterns that carry the hot fluid to the bottom, as shown in Figure 4 at e = 0.4 mm. Indeed, the formed hexagonal patterns govern the whole domain and extend through the ingot depth, preserving the same position, as explained schematically in Figure 1. 

The temperature gradient at deeper planes (e = 0.4 mm) is weaker compared to the free top surface. However, the situation may change after solidification when the solute is rejected and the latent heat is released. Consequently, the velocity magnitude at the free top surface is higher than at the bottom region (about 27-fold); this is mainly caused by Marangoni flow, which is initiated at the free surface where there is a larger temperature gradient than at the bottom. The present analysis of the flow pattern and temperature distribution offers valuable insights into the flow behavior and the formation of the hexagonal patterns in the presence of a free surface. This analysis provides a preliminary estimate of the segregation pattern formed during solidification. 

### 4.4. Marangoni Effect after Solidification

**Figure 5:**
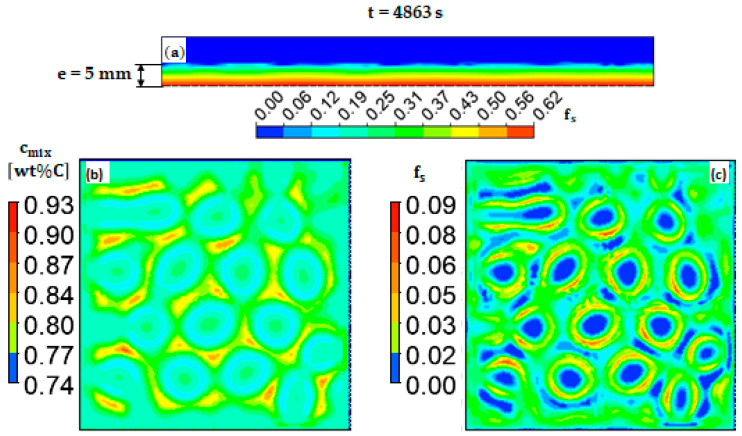
The calculated solid fraction after t = 4863 s at (**a**) a vertical cut plane (*yz* plane) and (**c**) a horizontally cut plane (*xz* plane). Also, see (**b**) for the mixture concentration at the liquid–solid interface of horizontally cut plane (*xz* plane).

In this section, the solidification of the alloy is allowed and the impact of the flow pattern on the segregation structure is investigated. Thus, a convection heat transfer boundary condition with a heat transfer coefficient (HTC) of $20\;W/m^{2}K$ was imposed along the free surface to govern the amount of heat transferred between the molten metal and the surroundings. A large heat transfer coefficient of $600\;W/m^{2}K$ was also applied to the bottom surface to promote the growth of the columnar grains from the bottom upward. The formed solid fraction and the carbon concentration in the mixture (segregation pattern) when the solidification front reached the middle height of the ingot (at e = 5 mm) after t = 4863 s is shown in Figure 5. The columnar dendrite trunks exhibit higher $f_{s}$ close to the cold bottom of the ingot and decreases gradually towards the hot top (Figure 5a). As expected, the solid fraction and the segregation structures formed at e = 5 mm above the ingot bottom have shapes analogous to the hexagonal convection cells formed at the free top surface. This behavior is mainly due to the Marangoni flow generated by surface tension, which is produced by the hexagonal structures throughout the liquid depth. However, a higher solute concentration was noted around the cells. 

The solid front (at e = 5 mm from bottom) exhibited a concave shape, as evident from Figure 5a, where periodic depressions in the solid front are observed. These depressions originate from the flow configuration generated by Marangoni flow, which drives the colder regions upwards. However, a downward hot flow persists in the center of the cells, maintaining these regions in liquid state, as depicted in Figure 5c (blue region). The segregation map (Figure 5b) gives the solute distribution, which varies in accordance with Equation (10). The region with higher solid fraction is characterized by a negative segregation pattern; this is because the mixture concentration is lower than the nominal value. Conversely, a positive segregation was developed around the cells as a result of the solute that was rejected from the solid to the liquid.

**Figure 6:**
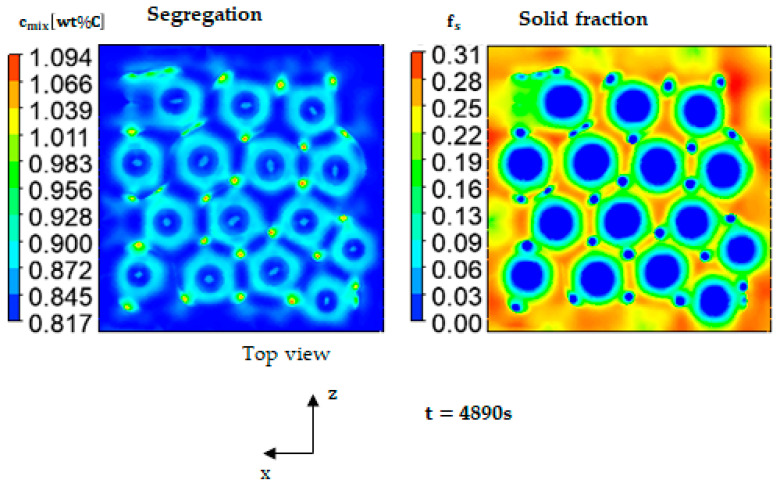
Mixture concentration (**left**) and solid fraction (**right**), presented at the level of the free surface.

The solidification front takes about 4863 s (calculation time) or 128 s (solidification time) to reach the mid-height of the ingot. As the solidification front advanced towards the top, the thermal gradient gradually decreased, leading to an accelerated solidification rate. Figure 6 presents top-view snapshots of the solid fraction (right) and the segregation map (left) at the final stages of solidification. The solidification front reached the top surface at t = 4890 s, confirming that the second half of the geometry solidified within only 27 s.

**Figure 7:**
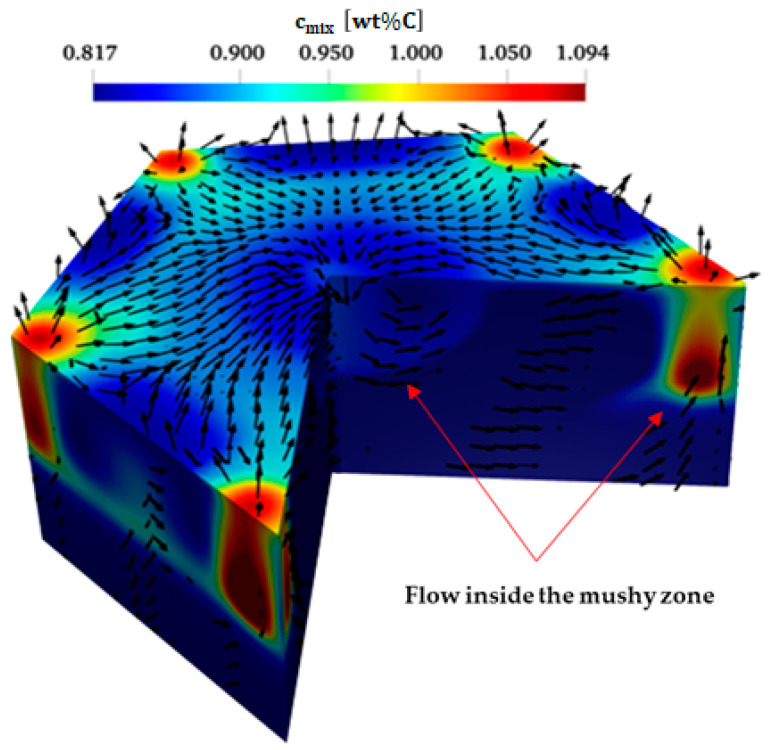
Three-dimensional zoomed-in view of one hexagonal cell showing the segregation and velocity vectors at the free surface and inside the mushy zone.

The columnar grains continue to solidify in a hexagonal cellular pattern, where the Marangoni flow is becoming more intense when the remaining liquid ahead of the solid front becomes shallower. However, the centers of the cells are still in a liquid state and characterized by higher temperature. It is noteworthy that the blue circular features of full liquid scattering at the periphery of the hexagonal cells are evident in Figure 6 (right). These circles are in the form of tubes extending inside the mushy zone and are characterized by a high carbon content (Figure 6 left). These tubes are called segregation channels, as shown in the zoomed-in view of a single hexagon (Figure 7). They may also play a role in governing the hexagons’ location thanks to the arising flow presented by the black vectors inside the segregated channels shown in Figure 7.

At the free surface, the upward stream inside the segregation channels flows toward the center of the hexagons (see Figure 7). This liquid stream recirculates downwards through the mushy zone from the center of the hexagon at the free surface and then again towards the segregation channels. The flow patterns at the free surface and within the mushy zone exhibit similar characteristics to those observed in the liquid state prior to solidification, as discussed in Section 4.2. This similarity arises from the influence of the positive coefficient of surface tension, which drives the flow’s recirculation. Therefore, the Marangoni flow induced by the surface tension gradient governs the configuration of segregation structures, particularly the location of the segregation channels.

**Figure 8:**
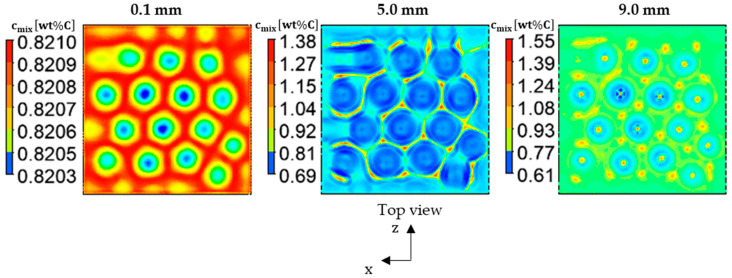
The contour of mixture concentration at the end of solidification of the Fe-0.82wt% ingot at various cut levels: 0.1 mm (**left**), 5 mm (**middle**); and 9 mm (**right**).

At the end of solidification ($f_{s}\approx 0.99$ in the whole domain), the segregation pattern at three different cut planes in the solidification ingot was calculated; representations of the 0.1 mm, 5 mm, and 9 mm cut planes are presented in Figure 8. It is obvious that the polygonised cells of the segregation patterns (very close to the hexagonal shape) are formed throughout the thickness of the ingot as a result of the Marangoni-induced flow. These segregation cells are characterized by higher carbon concentration at their edges compared to their centers. Also, the cells close to the bottom (0.1 mm plane) have thicker carbon-rich peripherals; however, the minimum and maximum segregation difference (ΔC) is weak. At the 5 mm cut plane, the regions with positive segregation become thinner, with a dramatic increase in segregation that reached 1.38wt%C. Close to the free surface (the 9 mm cut plane), very high positive segregation nodes are scattered around the hexagonal cells; their centers survived, even at the end of the solidification process. This is evidence for decaying segregated liquid recirculation, as explained in Figure 7.

**Figure 9:**
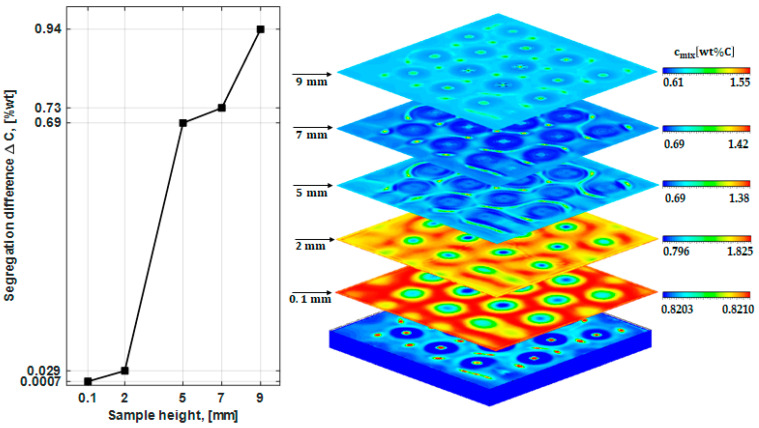
Segregation difference versus the sample (ingot) height at 0.1, 2, 5, 7, and 9 mm cut planes from the bottom to the top, respectively.

A quantitative analysis for the segregation that forms in different regions at the end of the solidification process was conducted, as shown in Figure 9. The segregation differences (noted as “ΔC”) at the 0.1, 2, 5, 7, and 9 mm cut planes were plotted (Figure 9, left) in comparison with the segregation contours (Figure 9, right). To facilitate the mapping between the left and right parts of Figure 9, the cut planes shown on the right present the minimum and the maximum segregation levels of each plane. The results reveal a significant increase in segregation differences, from ΔC = 0.0007 for the 0.1 mm cut plane to ΔC = 0.94-wt% for the 9 mm cut plane. A very weak segregation difference was observed close to the ingot’s bottom. This can be attributed to the weaker convection streams at the bottom compared to the top, as discussed previously in Figure 4 (weaker Marangoni flow at the bottom). As the solidification starts from the bottom and advances towards the top, a small concentration gradient and, consequently, a weak segregation are formed at the horizontal plane (e = 0.1 mm). However, at the e = 2 mm horizontally cut plane, the segregation difference sharply increased to 0.029-wt% (about 41-fold) due to the substantial solute gradient. With the same tendency, the segregation difference further increased to 0.69-wt% at the mid-height (e = 5 mm), which is 23-fold greater than the segregation difference at e = 2 mm. The segregation difference continued to rise with increasing height of the ingot, reaching 0.73 and 0.94-wt% at e = 7 mm and e = 9 mm, respectively, as shown in Figure 9.

The results presented in Figure 9 provide compelling evidence that Marangoni flow directly impacted the morphology and the level of the segregation that has formed throughout the ingot. Interestingly, it was observed that an even weaker Marangoni flow (near the bottom region) was able to produce a hexagonal segregation pattern; however, this had a smaller ΔC value. On the other hand, a stronger Marangoni flow, especially one that was closer to the free surface, was found to lead to more noticeable variations in the segregation.

**Figure 10:**
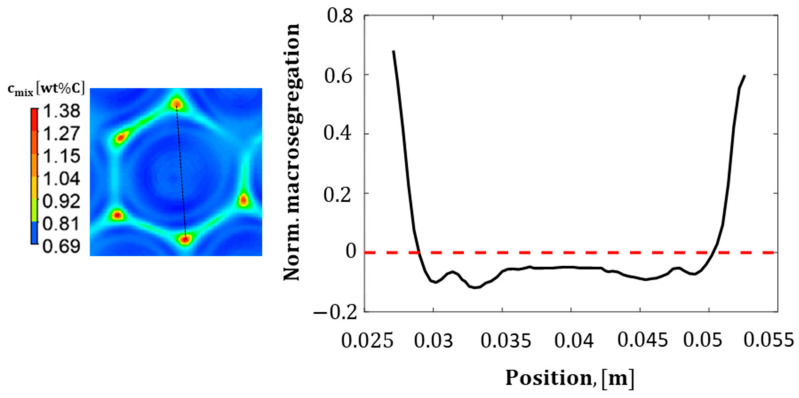
Normalized macro-segregation along the black line presented on the left picture in one hexagon obtained in the static case at e = 5 mm after full solidification.

The mechanical properties and the melting point of carbon steel are very sensitive to the steel’s carbon content. The carbon content within the solidification ingot was subjected to dramatic variations, as shown in Figure 8; it may reach ~1.6wt%C at certain segregation nodes. This value is very close to the those in the cast iron composition range. In these areas, a large amount of the hard and brittle ‘cementite’ micro-constituent may be formed; in contrast, the other carbon-depleted areas contain a more ductile phase, ’ferrite’. On the other hand, the C-rich areas exhibit lower liquidus temperatures that may cause partial melting during the subsequent heat treatment processes of steel. Furthermore, a structural failure is expected to occur at the brittle nodes during the subsequent deformation processes. Therefore, the current analysis may assist researchers and industrial developers in building an understanding of the origins of segregation in depth; this is the key to avoiding or minimizing its formation. An area plot of the positive/negative segregation values in terms of normalized macro-segregation (cmixc0−1) [24] within one convection cell is presented in Figure 10, with the initial alloy composition being given by the value of 0; in contrast, positive and negative macro-segregations are being given by values above or below zero, respectively [24]. The result presented in Figure 10 (on the left) shows the contours of the mixture’s concentration. The carbon-rich areas are highlighted in red; meanwhile, the carbon-depleted regions are shown using blue. Figure 10 (on the right) illustrates the normalized macro-segregation along the black dashed line drawn through the hexagonal cells, as per Figure 10 (left). The results indicate a positive segregation at the edge of the hexagonal cell and a fluctuating negative segregation in the inner region of the hexagon.

In the future, the authors intend to extend the findings of the current study to research the effects of the temperature gradient throughout the height of the mold on the formed segregation. In attempts to eliminate the segregation driven by the Marangoni effect thus far, the authors are yet to explore the application of surface tension modification fluxes to the free surface and superimposing an electromagnetic stirring field.

## 5. Conclusions

A 3D mixture numerical simulation model was developed for the solidification of an Fe-0.82wt%C alloy under the effect of a Marangoni flow generated by the surface tension at the free surface. The finite volume method was used. The model succeeded in exploring the coupled effect of Marangoni flow and buoyancy convection on the velocity and the thermal fields. In addition, the impact of the Marangoni flow on the morphology of the solidification front and especially of the segregation structures was investigated. The results demonstrated that the Marangoni flow effectively generated polygonised/hexagonal convective cells at the free surface, with this effect extending through the liquid depth. The size and the number of the hexagonal patterns were found to be directly affected by the liquid thickness and the thermal gradient. Increasing the thermal gradient led to a rise in the number of cells, while increasing the ingot height resulted in larger hexagons. The temperature coefficient of surface tension was identified as a key factor determining the flow direction at the free surface. A positive value, indicating that the increase in surface tension occurred with the increase in temperature, promoted a radial inward flow within the cells. Moreover, the Marangoni flow had significantly impacted the temperature field by transporting hot fluid to the bottom, facilitating the formation of hexagonal cell structures. The solidification started from the bottom and moved upward due to the intense cooling applied to the bottom surface. The growing solidification front exhibited a concave morphology, as the hottest regions are located at the centers of the cells. After the full solidification, the segregation was found to be in perfect hexagonal shapes. However, stronger segregation was formed close to the free surface where the Marangoni flow was is effective. This study illuminates the crucial role of surface-tension-driven Marangoni flow in producing hexagonal flow patterns before and during the solidification process. It provides valuable insights into the complex interplay between Marangoni flow, buoyancy convection, and solidification phenomena, and introduces a deeper understanding of the origins of the segregation patterns for various industrial applications. The authors plan to conduct further study into suppressing the contribution of Marangoni flow to macro-segregation.

## Figures and Tables

**Table 1:** Thermo-dynamic/physical properties of Fe-0.82wt%C steel.

Property/Parameters	Symbol	Unit	Value	Ref.
Nominal concentration of the alloy	$C_{0}$	wt%C	0.82	[21]
Liquidus temperature	$T_{liq}$	K	1745.15	Present work
Melting temperature	$T_{m}$	K	1809	[21]
Partition coefficient	k	*-*	0.34	[21]
Liquidus slope	$m_{l}$	Kwt%	−78	[21]
Density	$\rho_{l},\rho_{s}$, $\rho_{0}$	$kg/m^{3}$	7020	[21]
Dynamic Viscosity	μ	$m^{2}/s$	${5.5\times 10}^{-3}$	[21]
Liquid thermal Conductivity	λ	WmK	33	[21]
Heat Capacity	$C_{p,l}$	JkgK	824	[21]
	$C_{p,s}$		648	[21]
Solutal expansion coefficient	$\beta_{c}$	${wt}^{-1}$	0.011	[22]
Thermal expansion coefficient	$\beta_{T}$	$K^{-1}$	0.0002	[22]
Latent heat	L	Jkg	${2.72\times 10}^{5}$	[21]

**Table 2:** Configuration of the 3D grids used in various simulation case studies.

	No. Grid Elements	Liquid Depth “e”, mm	Ingot Length “L”, mm	Thermal Gradient, K
Case (a)	1,629,350	10	100	65
Case (b)	203,125	1.25	12.5	100
Case (c)	203,125	1.25	12.5	65

## Data Availability

Data are contained within the article.
